# Where We Go From Here: Harnessing Queer Perspectives to Advance Practice in Ecology and Evolutionary Biology

**DOI:** 10.1002/ece3.73063

**Published:** 2026-03-17

**Authors:** Madeline G. Eppley, Robert J. Dellinger, Leila Curtis, Cesar O. Estien, Lindsey Forg, Angela J. Jones, Ally Swank, Andy Lee

**Affiliations:** ^1^ Department of Marine and Environmental Sciences Northeastern University Marine Science Center Nahant Massachusetts USA; ^2^ Department of Oceanic & Atmospheric Sciences University of California Los Angeles California USA; ^3^ Department of Environmental Science, Policy, and Management University of California Berkeley California USA; ^4^ Department of Biology Boston University Boston Massachusetts USA; ^5^ Department of Biological Sciences Purdue University West Lafayette Indiana USA

**Keywords:** bias, equity and inclusion, philosophy of science, queer ecology, science policy, STEM education

## Abstract

In this manuscript, we invite the field of ecology and evolutionary biology (EEB) to interrogate our relationship with society at large, consider the perspective of queer scientists, and take action to produce more accurate and objective science. Science and society continuously shape one another; this relationship determines what science is funded, which inquiries are pursued, and whose voices are included in the production and dissemination of knowledge. In EEB, many scientists have worked to address biases that originated from and were legitimized by societal norms. Here, we investigate this science‐society relationship further by exploring how this relationship has historically impacted and continually impacts LGBTQ+ people. Using illustrative examples, we discuss societal trends and their influence on science over time, current biases in interpreting data, and the downstream impacts on policy. To move beyond inclusion to an epistemological intervention, we propose a framework that expands the conceptual boundaries of what is considered scientific knowledge and actively considers the political dimensions of research inquiries. We draw on our queer lived experiences to outline six principles to confront existing biases and produce better science: (1) recognize intersections between systems of oppression; (2) incorporate queer knowledges into scientific thought; (3) challenge biased (e.g., binary, deterministic) frameworks; (4) understand that language and hypothesis framing shape both science and its societal interpretation; (5) embed ethics and political responsibility in the development of research inquiries; (6) accept that we do not need answers for everything. To implement these principles, we provide guidance for individual and collective actions in the classroom, academic societies, and research. By integrating these principles within mainstream scientific practice, we can strengthen the reciprocal relationship between science and society, where advances in one transform the other, collectively moving towards knowledge that is more accurate, reproducible, and inclusive.

## Graphical abstract

Science and society continuously shape one another; this relationship determines what science is funded, which inquiries are pursued, and whose voices are included in the production and dissemination of knowledge. Here, we draw on our queer lived experiences to outline six principles to confront existing biases and produce better science: (1) recognize intersections between systems of oppression; (2) incorporating queer knowledges into scientific thought; (3) challenge biased (e.g., binary, deterministic) frameworks; (4) understand that language and hypothesis framing shape both science and its societal interpretation; (5) embed ethics and political responsibility in the development of research inquiries; (6) accept that we do not need answers for everything. To implement these principles, we provide guidance for individual and collective actions in the classroom, academic societies, and in research. By integrating these principles within mainstream scientific practice, we can strengthen the reciprocal relationship between science and society, where advances in one transform the other, collectively moving towards knowledge that is more accurate, reproducible, and inclusive.

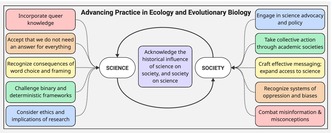

*‘Queer’ not as being about who you're having sex with (that can be a dimension of it), but ‘queer’ as being about the self that is at odds with everything around it and that has to invent and create and find a place to speak and to thrive and to live*.

― bell hooks

## 1 Introduction

To be queer is to inhabit a world at odds with queerness, and to respond by inventing new ways of knowing, surviving, and being. Science, too, has long been conducted in ways that are at odds with the natural systems it seeks to describe. Historically, for example, ecology and evolutionary biology (EEB) has often framed nature through a reductionist lens with binary classification rather than describing its true complexity (e.g., Box 1). This is because science does not emerge separately from the world it seeks to describe; rather, it is co‐produced through historical, political, and economic structures that influence what questions are asked, how evidence is interpreted, and which narratives are legitimized (Lewontin 1996). Thus, to address biases, EEB must invent and create ways of practicing science that expand the possibilities of how we study life. In this manuscript, we invite the field of EEB to examine our relationship with society at large, consider the perspective of queer scientists, and take action to produce more accurate and objective science.

BOX 1 **Case studies of bias in scientific interpretation.**

Although scientific observation and data collection can be objective, interpretation and reporting are influenced by societal norms which are co‐produced through historical, political, and social structures. Some of the biases that we explicitly confront in this paper include:

- *Reductionism*: Bias that results from the simplification of complex topics into smaller parts that are then representative of the whole.
- *Determinism*: Bias that results from the idea that biological traits (e.g., queerness) are primarily or exclusively caused by genetic factors and are therefore hardwired and immutable.
- *Essentialism*: Bias that results from thinking that identity (e.g., gender) is innate.
- *Binary thinking*: Bias that results from a tendency to classify things into two categories, ignoring nuance, complexities, and exceptions that might exist.

The following are instances of societal biases that have influenced scientific practice (for further examples, see Cronin et al. 2021 and Anderson et al. 2024).

**Case 1: Female birdsong dismissed until investigated by female researchers**

Female birdsong had been systematically overlooked in studies of avian sexual selection because of androcentric (male‐centered) bias influencing scientific interpretation. The prevailing paradigm considered male birds as performers and female birds as passive choosers in sexual selection. This bias impacted the understanding of female birds' active roles in communication and sexual selection until female researchers entered the field, bringing different perspectives that allowed for a paradigm shift (Haines et al. 2020; Ah‐King and Ahnesjö 2013).

**Case 2: Primary morphology of females in well‐studied species is overlooked**

Androcentric research bias has deemed female reproductive structures as less important, causing researchers to routinely overlook basic anatomical features (Folwell, Sanders, and Crowe‐Riddell 2022; Folwell, Sanders, Brennan, and Crowe‐Riddell 2022). Snake anatomy has been well studied for centuries, but the female reproductive structure, hemiclitores, remained undiscovered until very recently (Folwell, Sanders, and Crowe‐Riddell 2022; Folwell, Sanders, Brennan, and Crowe‐Riddell 2022). The recent nature of this discovery demonstrates how entrenched these biases are.

**Case 3: Non‐reporting bias in species exhibiting same‐sex behaviors**

Field biologists regularly document and photograph pairs of gulls engaging in courtship displays, nest building, and chick rearing. However, when these behaviors occur between same‐sex pairs (image below), the biologists may (1) fail to record these observations in their field notes, (2) dismiss the behavior as “practice,” “mistakes,” or “abnormal behavior”, or (3) exclude the individuals from data analyses and published reports (Anderson et al. 2024).

This non‐reporting bias creates a descriptive circularity problem where same‐sex behaviors appear rare in the scientific literature (because they are systematically underreported), which reinforces the assumption that they are rare or insignificant (further justifying non‐reporting). Exacerbating the bias, when same‐sex behaviors are documented, they are often treated as anomalies rather than as evidence that challenges existing paradigms (Anderson et al. 2024). This systematic blind spot stems from a heteronormative bias; frameworks built on this bias assume reproductive fitness is exclusively achieved through male–female pairings, causing researchers to overlook, misinterpret, or devalue same‐sex behaviors even when directly observed. Consequently, the prevalence and ecological significance of same‐sex behaviors across hundreds of species remain understudied and underreported despite their regular occurrence in nature.

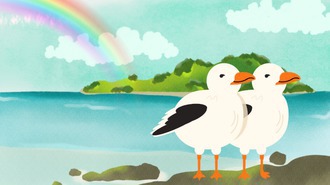

**Practical example: Binary sex categories inaccurately classify hermaphroditic individuals**

In this practical example, ecologists are collecting data on the gonad tissue and condition of the hermaphroditic eastern oyster ( *Crassostrea virginica* ). When examining tissue under a microscope (illustration below), cloudiness (sperm, top), small circular structures (eggs, middle), or both (bottom) can be seen . If scientists examine an oyster with both eggs and sperm present, they may : (1) remove it from the study, (2) misclassify it as either male or female, or (3) fail to recognize the hermaphroditic state altogether. In any of these cases, the societally influenced sex binary can lead to biased or inaccurate reporting and lack of reproducibility (Eppley et al. 2026).

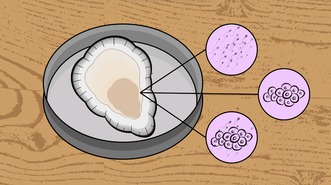

Science and society do not operate in isolation; they shape and re‐shape each other in a dynamic and reciprocal process. As Lewontin (1996) states, “Scientists do not begin life as scientists, after all, but as social beings immersed in a family, a state, a productive structure, and they view nature through a lens that has been molded by their social experience”. In this framework, science is not merely shaped by society; it also shapes the society in return through the frameworks it constructs, the knowledge it circulates, and the power it authorizes (Lubchenco 1998; Rozzi 1999). Although ecologists and evolutionary biologists have worked to expose and address many historic biases in our research that were, at the time, reflective of societal influence, we now confront the question of how this science‐society relationship has historically impacted and continues to impact the queer community (including lesbian, gay, trans, queer, intersex, asexual, two spirit, and other identities not explicitly listed; hereafter written as LGBTQ+).

We are driven by the urgent need to counteract harmful misrepresentations (e.g., misinformation (false or misleading), disinformation (false or misleading; intent to deceive), and malinformation (false or misleading; intent to cause harm)) of sex and gender used to fuel anti‐LGBTQ+ policy and by our unique perspective as both objects of scientific inquiry and as scientists ourselves (Box 2). As queer scientists in EEB, we propose actively queering ecology and evolution to advance better science—by reducing bias in data interpretation, avoiding circular reasoning in scientific description, facilitating reproducibility, and improving the recruitment and retention of queer scientists. Our vision of queering ecology and evolution includes six principles: (1) incorporates queer knowledge into scientific thought, (2) challenges biased (e.g., binary, deterministic) frameworks, (3) chooses inclusive terminology and understands that hypothesis‐framing matters, (4) considers ethics and political implications when developing lines of research inquiry, (5) recognizes the intersection between systems of oppression, and (6) realizes that not all questions need answers (see *Where we go from here: Queering ecology and evolutionary biology*) (Chen 2012; Estien 2025; Ferdinand 2021; Freyne 2020; D. J. Haraway 2016; Packer and Lambert 2022; Seymour 2017; Trisos et al. 2021).

In this paper, we provide a roadmap for queering EEB. We start by investigating the historical roots of the reciprocal relationship between science and society, noting the recurring societal biases that have shaped the field over time (e.g., racism, sexism). We then draw clear parallels to the contemporary landscape, where societal, social, and political priorities shape the future of science by dictating scientific funding priorities. By reviewing historic trends alongside the contemporary landscape, we illuminate how past dynamics continue to influence present practices. We then describe the current social and political landscape of science funding and document the extent of impacts on the field of EEB and the queer community. Finally, we outline our vision and framework for queering EEB, connecting each recommendation to concrete and actionable items for scientists and scientific societies.

BOX 2 **Current social and political landscape and impacts on LGBTQ+ people and scientists in the US.**

**Societal impacts on LGBTQ+ people**

LGBTQ+ rights, and specifically trans rights, are under intense scrutiny in the current social and political landscape within the US. Political leaders have successfully campaigned on reducing LGBTQ+ rights, and their policies have caused direct harm and elevated discrimination towards the LGBTQ+ community (Sudai et al. 2022). These policies include but are not limited to state‐ and federal‐level restrictions on gender‐affirming healthcare, denied bathroom access, refusal of correct pronoun usage, and exclusion from participation in amateur and collegiate sports (Brightman et al. 2024; Ha et al. 2014; Redfield et al. 2024).

US lawmakers introduced more than 500 anti‐LGBTQ+ bills in 2024, nearly three times as many as the year prior (ACLU 2024a; Funakoshi 2023; Miyagi et al. 2021). Of these, over 140 bills aim to restrict gender‐affirming healthcare for transgender, gender non‐conforming, and intersex (TGNCI) individuals, and 27 criminalize providing, assisting, or undergoing gender‐affirming care as a felony, carrying a sentence of up to life in prison (Funakoshi 2023).

Given these attacks on the LGBTQ+ community (ACLU 2024b; Esseks 2018; Human Rights Campaign 2024), calls to mental health crisis lines from LGBTQ+ youth increased by 700% following November 5th, 2024, the most recent US presidential election (Trevor News 2024). Although tremendous progress has been made for LGBTQ+ rights over recent years, there is grave concern for the regression of LGBTQ+ lives and rights in 2025 and beyond, emphasizing the need to understand how LGBTQ+ people can be better protected and supported.

**Societal impacts on LGBTQ+ scientists**

The negative effects of the current US social and political landscape impact many aspects of health, wellbeing, and success of the LGBTQ+ community, as seen through the anonymized testimonies of LGBTQ+ EEB scientists (image below). It has been well‐documented that LGBTQ+ scientists who are subject to discrimination, harassment, and microaggressions have a higher likelihood of leaving academia (Boustani and Taylor 2020; K. A. Taylor 2021), and the majority of these instances occur in jurisdictions without policies in place to protect LGBTQ+ communities from such behaviors (Truszczynski et al. 2022). Unsafe workplace environments can lead to non‐disclosure of queer identities (Beagan et al. 2022; Kelly et al. 2021; Miller et al. 2019; Willis 2011), which is directly linked to reduced contributions, leadership, and publication rates of queer scientists (Nelson et al. 2022). Recent large‐scale analyses show that LGBTQ+ biologists, particularly TGNCI individuals, report lower belonging, increased exclusionary experiences, and decreased morale across academic and professional biology settings (Cooper et al. 2025). These challenges are also arising at a time when science is more widely available than ever in the contemporary digital landscape (Huber et al. 2019). To take advantage of opportunities for career growth, scientists are increasing their presence digitally and on social media (e.g., X formerly known as Twitter; Bluesky). However, scientists of marginalized identities are often subjected to volatile interactions online, including hateful comments, trolling, and negative news coverage—all of which impose stress on mental wellbeing and can ultimately contribute to lack of retention in the field (Marchi et al. 2024; Nadal et al. 2014; Sears et al. 2024).

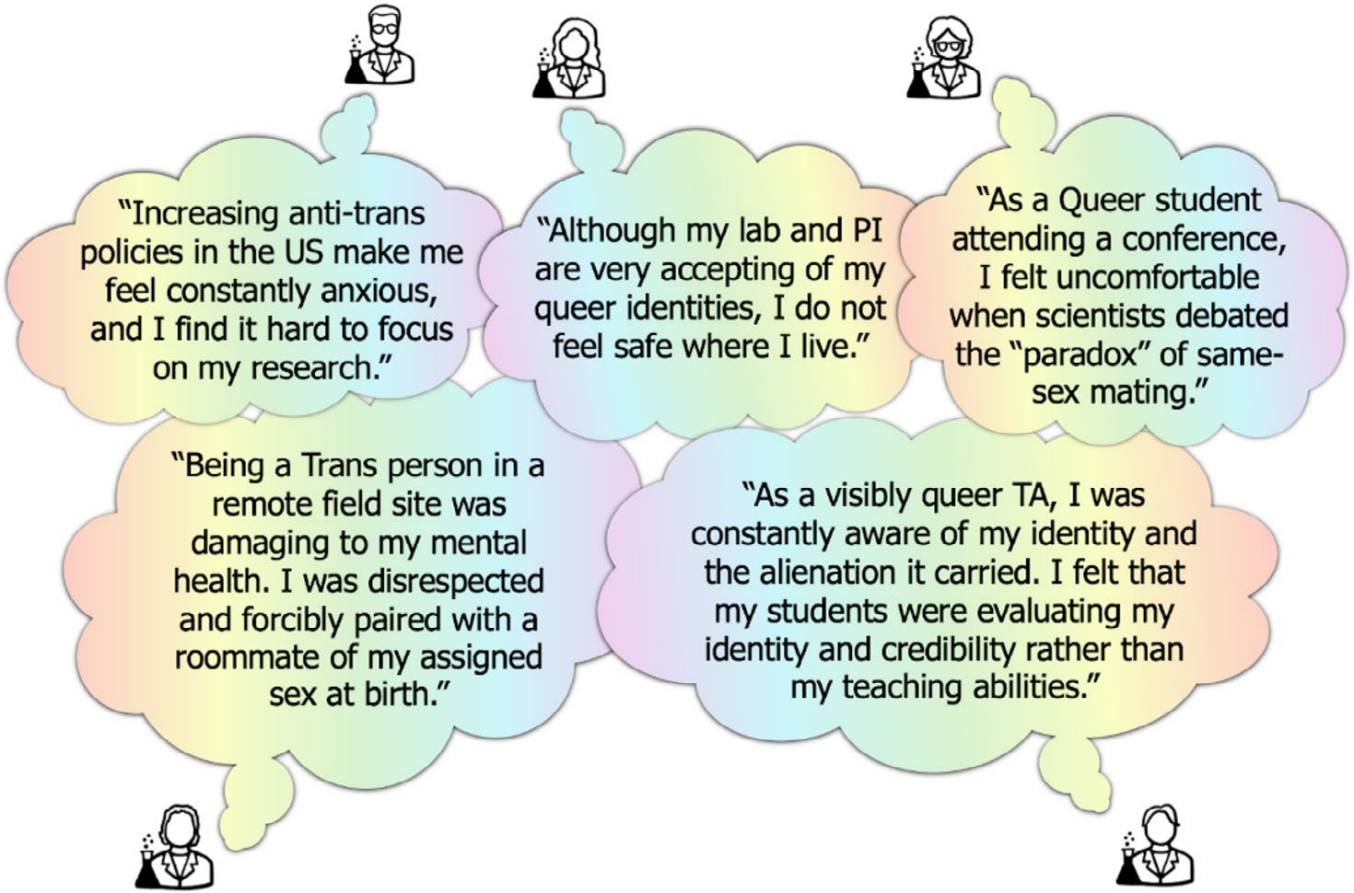

**Anonymized testimonies regarding the impacts of the current US social and political landscape and within‐EEB climate on LGBTQ+ scientists and students**

Although we focus on the plight of the LGBTQ+ community in this manuscript, we recognize that these harmful political sentiments and resulting policies did not arise in a vacuum, nor does it impact all LGBTQ+ people equally. For example, non‐white LGBTQ+ people face higher levels of discrimination than white LGBTQ+ members (Casey et al. 2019; Whitfield et al. 2014), and TGNCI individuals face elevated discrimination compared to non‐trans queer people (Hoxmeier and Madlem 2018; Lombardi et al. 2001; Sears et al. 2024). In particular, trans women of color face higher rates of fatal violence and discrimination than most other minorities (Freeman 2018; Human Rights Campaign Foundation 2017). In addition to anti‐LGBTQ+ legislation, racist and anti‐immigration agendas have also increased over the recent years (Kuroki 2021; Lajevardi and Oskooii 2018; Lies et al. 2024). For members of the LGBTQ+ community who hold multiple marginalized identities, including race, ethnicity, and disability, these issues are even more pertinent as marginalized identities compound and interact with each other (intersectionality, sensu Crenshaw 2013).

## 2 Science and Society

Across the lifespan of an organism, genes, phenotype, and environments interact dynamically, shaping ecological interactions and evolutionary trajectories. In Levins and Lewontin's constructivist view, organisms are simultaneously subjects and objects of evolutionary and ecological change; they are shaped by their environments even as they actively transform those environments through their activities (e.g., niche construction) (Lewontin and Levins 1997). Ecological and evolutionary outcomes, therefore, do not arise solely from external conditions but emerge from relational processes that span across generations (Lewontin 1983b; Lewontin and Levins 1997).

The co‐constructive model parallels the deeply interdependent relationship between science and society. Just as organisms transform their environments in ways that reshape evolutionary trajectories, scientific practice reshapes the material and ideological conditions under which knowledge is produced while simultaneously being shaped by those same conditions. Societal priorities and norms shape scientific interpretation, what science is funded, which inquiries are pursued, and whose voices are included in the production and dissemination of knowledge, processes that are never neutral but reflections of power (D. Haraway 2013; Trisos et al. 2021; van Eck et al. 2024). In turn, scientific discoveries shape cultural narratives, policy, and social institutions, influencing how society conceptualizes the natural and physical world. Queering ecology and evolutionary biology builds on the co‐constructive model: like the reciprocal interactions between organisms and environments, science and society co‐produce the meanings of sex, gender, and biology, shaping both research trajectories and the lived realities of queer and trans people (Chen 2012; Trisos et al. 2021). For scientific thought to be comprehensive and expansive, it must include perspectives from diverse lived experiences that span race, ethnicity, sex, gender, sexuality, and other identities.

Social, societal, cultural, and political dynamics have long shaped the trajectory of science, influencing not only research priorities but also how we perceive and understand the natural and physical world. For instance, interest in weapon development during World War II led to the Manhattan Project, which received $2 billion in federal funding between 1942 and 1945 (Lubchenco 1998), establishing premier national laboratories such as Los Alamos Laboratory in New Mexico (Los Alamos National Laboratory 2025), Oak Ridge National Laboratory in Tennessee (ORNL Review 2018), and Lawrence Livermore National Laboratory in California (LLNL 2025). Post‐war, these weapons development facilities were transformed into permanent federally funded research centers and National Historic Parks that now employ thousands of scientists across disciplines (Los Alamos National Laboratory 2025; ORNL Review 2018). Resulting technologies produced during this era fundamentally transformed international relations, military strategy, and energy production while establishing a model for government‐funded science that continues to shape research directions today.

Later, in the space race era, funding for science increased in an unprecedented manner (Lubchenco 1998; J. Mervis 2021). During this time, Congress passed the National Defense Education Act, investing $1 billion in science education, and NASA's budget skyrocketed from 1958 to 1965, representing 4.44% of the federal budget at its peak (today it represents just 0.4%) (Conn 2020). Environmental activism similarly transformed funding priorities, with the 1970 establishment of the Environmental Protection Agency (coinciding with the National Science Foundation's (NSF) first dedicated environmental research programs). The NSF budget for ecology and environmental biology grew from $54 million in 1970 to $352 million by 1980. Science during this period captivated national imagination: it revolutionized computing, launched widescale environmental monitoring, and transformed science education.

As this history shows, US scientists have benefited from relatively stable bipartisan support for research funding since the establishment of federal‐level science funding. Although funding has ebbed and flowed over time, the general trajectory has remained positive. In fact, the NSF has survived amidst decades of changing political environments and navigated through periods of political scrutiny while maintaining a stable core mission (NSF 2025).

Unfortunately, this historic stability is eroding under an unprecedented lack of political and societal interest in funding science. The current administration's proposed budget cuts for fiscal year 2026 (FY26) exceed 50% for federal science institutions like the NSF, National Oceanic and Atmospheric Administration (NOAA), and the National Institute of Health (NIH), signaling a dramatic shift in science policy priorities. Uncertainty across scientific disciplines has increased as institutions experienced recent mass layoffs, budget cuts, and systematic restructuring (American Society of Naturalists 2025; EPA 2025; Gewin 2026; Heidt 2025; Ro 2025). Further demonstrating this pattern, the Trump administration canceled US science agencies' subscriptions to Springer Nature journals, citing concerns over editorial bias (Brainard 2025).

The US is also investing less and less into science (measured as a share of national GDP, NCSES 2024), whereas other countries are increasing their investments. Following the same pattern, the contribution of business funding to total Research & Development (R&D) in the US has increased significantly over time, with the federal government's share of basic research declining from about 60% in 2000 to 40% in 2022, whereas business funding rose from 20% to 37% over the same period (NCSES 2024), thereby positioning private interests to exert greater influence over which scientific inquiries are pursued more than ever before. This declining public investment threatens the ability of the US to prepare for and respond to emerging infectious diseases, maintain global competitiveness in artificial intelligence and advanced computing, and address conservation challenges that threaten food security in agriculture and aquaculture systems, among many other concerns.

Despite these constraints, scientists retain significant power to shape social and political perceptions through their research priorities, methodological choices, and public engagement. Scientists have always strived to influence society (e.g., as in Roughgarden 2004; Lewontin 1983a, 1983b), and this effort continues in the contemporary landscape. For instance, effort has been made to standardize inclusive scientific terminology (Rice et al. 2025) and introduce anti‐racist interventions (Cronin et al. 2021), both of which redirected attention towards overlooked issues of social importance. This responsibility becomes particularly crucial when examining how social inequities themselves shape the environments we study and our understanding of them.

Society (and social inequities) also structures the physical and biological environment, who is positioned to observe and interpret them, and thus our scientific understanding of those landscapes. For example, systemic racism and classism have reconfigured urban environments (Schell et al. 2020), creating stratified patterns of structural inequality (e.g., green cover, impervious surface cover, environmental pollutants, etc.) that, in turn, shape patterns of wildlife presence, species interactions, and genetic diversity (Carlen, Caizergues, et al. 2025; Estien et al. 2024; Hentati et al. 2025; Wilkinson 2023; Wood et al. 2024). These inequities also influence which ecological harms are studied and addressed (Gadsden et al. 2022): pollution research, for instance, long failed to examine disproportionate impacts on marginalized communities until environmental justice movements forced scientific attention to these issues (Bullard 1993; Mohai and Saha 2015; D. E. Taylor 2020). Societal biases also influence the biological questions that are asked and the behaviors that are documented. For example, same‐sex behaviors are widely observed in nature, yet remain under‐documented in the literature because of bias in scientific practice (e.g., Box 1) (Anderson et al. 2024). Even our understanding of genetic diversity and biodiversity has been distorted by colonial legacies and socio‐economic inequities, as research is largely driven by high‐income countries. This bias not only creates dependency on external expertise but also introduces structural sampling biases that skew our understanding of biodiversity in underrepresented regions (Hughes et al. 2023; Linck and Cadena 2024; Raja et al. 2022). Collectively, these examples highlight how societal, social, and political dynamics not only determine which scientific inquiries receive funding but also fundamentally shape the knowledge produced across space and time. Studies that directly address these structural inequities empirically can help us break down this cycle and recognize how deeply social, societal, and political systems shape both our physical environments and the knowledge we produce about them (Anderson et al. 2024; Bullard 1993; Carlen, Caizergues, et al. 2025; Estien et al. 2024; Gadsden et al. 2022; Hentati et al. 2025; Hughes et al. 2023; Linck and Cadena 2024; Mohai and Saha 2015; Raja et al. 2022; D. E. Taylor 2020; Wood et al. 2024).

### 2.1 Historic Relationship Between Society and EEB

It is impossible to examine the foundations of EEB without discussing how societal notions of human differences—particularly race, gender, class, ability, and sexuality—have long shaped how EEB scientists think and what research is pursued (Kamath et al. 2022). Thus, confronting the intertwined histories of EEB research on LGBTQ+ and Black, Indigenous, and People of Color (BIPOC) people allows us to remediate it (Baldwin 1998; Miriti et al. 2020).

Although race lacks a biological foundation (Lewontin 1972), the notion of race profoundly shaped early scientific thought. Carl Linnaeus's *Systema Naturae* (1735) formalized taxonomies that ranked human populations by skin color, behavior, and perceived morality, establishing racial typologies within the same classificatory system used for plants and animals (Subramaniam and Bartlett 2023). Nearly a century later, Darwin developed an evolutionary theory of sexual dimorphism (Somerville 1994), proposing sexual reproduction as a “testing ground of racial character” and “a causal force that could create new races” (Sheldon 2021). The roots of this bias can be traced to the pseudoscientific studies of African women's anatomy by European naturalists during the 18th and 19th centuries (Cuvier 1817; Flower and Murie 1867; Qureshi 2004). These studies, epitomized by the invasive and exploitative examinations of Saartje Baartman (commonly referred to by the racist epithet “Hottentot Venus”), framed African women as “primitive” through their physical characteristics, reinforcing racial boundaries and hierarchies (Flower and Murie 1867; Qureshi 2004). Many of these early studies were methodologically flawed and falsified, driven by preconceived racial hierarchies rather than empirical objectivity (Gould 1978, 1987). Building on these racialized frameworks, naturalists then formalized distinctions between racial groups, aligning them with ideas of morality, civilizational heirachy, and gender roles (Kampourakis and Peterson 2023).

Charles Darwin's evolutionary theories also emerged within this intellectual and cultural milieu, when he argued that natural selection led to an increasing divergence between the binary sexes as a marker of evolutionary progress towards being more “evolved” and “civilized” (Somerville 1994). Under this framework, white individuals were described as displaying the most dimorphism between sexes, and in comparison, individuals from non‐white ethnic groups were viewed as closer to the “primitive” state of “hermaphroditism” (McWhorter 2010; Somerville 1994). Later, Darwin asserted that sexual selection explained why male and female organisms adhered to nearly universal behavioral templates, which laid the foundation for evolutionary biology's first theory of gender and the enduring construction of biological essentialism and genetic determinism (Lewontin 1983a; Roughgarden 2004). These frameworks asserted that genetic predisposition to traits that lead to variation in sexuality is hardwired and immutable, and these traits then determine identity and gender.

Darwin's conception of natural selection was also deeply informed by the political economy of his time, for example, by Thomas Malthus's *Essay on the Principle of Population* (1826) and the competitive individualism of industrial capitalism. These ideas shaped his conceptualization of natural selection through metaphors of scarcity, struggle, and reproductive competition (Egerton 2011; Rozzi 1999; Young and Young 1985) and later informed social Darwinism and the eugenics movement, providing ideological foundations for the “survival of the fittest” that resurfaced in 20th‐century works like Herrnstein and Murray's *The Bell Curve* (1994). The resulting “survival of the fittest” paradigm, though not explicitly aimed at explaining sexuality, nevertheless shaped evolutionary biology's treatment of same‐sex behavior as “paradoxical”. If natural selection optimizes reproductive fitness, how could non‐reproductive sexual behaviors persist across species? This supposed paradox reflects not an inherent evolutionary puzzle but a heteronormative and reproduction‐centric bias in definitions of fitness and adaptation. Addressing these biases requires not only new empirical data but also the recovery of suppressed scientific traditions and a fundamental rethinking of assumptions inherited from society's colonial, eugenic, and heterosexist origins.

Although alternative ways of knowing have always existed within EEB, social and political forces have historically suppressed or erased observations of biological and sexual diversity. In contrast to Darwin's emphasis on competition, Peter Kropotkin's *Mutual Aid: A Factor of Evolution* (1902) highlighted cooperation as a central evolutionary force, documenting behaviors such as communal nesting, cooperative hunting, and collective care for young in animal and human societies to argue that mutual support enhanced survival and challenged strictly competitive views of evolution (Kropotkin and Huxley 2021). Despite its empirical grounding, *Mutual Aid* was long dismissed within mainstream evolutionary biology as overly idealistic and politically motivated, leading to its marginalization in favor of competition‐centered interpretations of natural selection (Gould 1988). Likewise, early 20th‐century research at the Berlin‐based Institut für Sexualwissenschaft (1919–1933) undertook pioneering studies of sexual and gender diversity in humans and non‐humans, but by 1933 Nazi authorities raided the institute and publicly incinerated its archives and library, effectively erasing decades of scholarship (Stryker 2017).

Historical erasure and exclusion of LGBTQ+ people is a global phenomenon institutionalized through both legal and scientific systems. The US repressed queer identities through domestic policy during the Lavender Scare (1950s–1970s), when LGBTQ+ people were barred from employment in government and scientific agencies, including the USDA, NASA, and the Department of Defense; at the same time, the US pressed its allies to adopt similar measures against the LGBTQ+ community (Alexander et al. 2024; Johnson 2023; Shibusawa 2012). Between 1953 and 1975, an estimated 5000–10,000 federal employees and scientists lost their jobs (Alexander et al. 2024; Faderman 2015; Johnson 2023). Even scientists who managed to remain in their fields, like Rachel Carson, were compelled to erase evidence of queer relationships to avoid professional ruin—concealing until recently how her love for Dorothy Freeman helped shape the multispecies ways of knowing foundational to *Silent Spring* (Maxwell 2017, 2025). These intertwined forces of political repression and self‐preservation erased generations of potential queer contributions to ecology and evolutionary biology.

Institutional barriers like those described above shape who is allowed to do science and consequently shape the kind of ecological and evolutionary knowledge produced. Documentation of same‐sex behavior in nature has long existed within ecological research yet was often censored or dismissed. As early as 1910, Dr. George Murray Levick documented same‐sex behavior in Adélie penguins during an Antarctic expedition, though his observations were censored from the 1915 official scientific records (Russell et al. 2012). Notes of male–male sexual behavior in captive bottlenose dolphins were recorded in 1948 (McBride and Hebb 1948), but these observations were dismissed as not relevant under wild conditions. Despite these and many other early examples, societal bias continued to influence the ways in which same‐sex behaviors were understood and presented, with publications pejoratively describing same‐sex behaviors using terminology such as “aberrant behavior” (Franz Sauer 1972), “unusual reproductive associations” (Conover et al. 1979), and “effeminate male behavior” (Berger 1985).

Although ecological research on same‐sex behaviors gained more visibility in the 1970s, it still remained contentious. A 1972 study on the nesting behavior of seagull colonies found that nearly 14% of all mated pairs on Anacapa Island, California were female–female mate pairs (Hunt and Hunt 1977). The discovery of same‐sex seagull mate pairs caused substantial public backlash, and a session of Congress in 1978 halted all funding for the NSF for 10 days with threats to cut further funding for biological behavioral research if additional seagull research was approved (Zhao 2021). Knowing that public perception curbed funding for ecological research, reports of same‐sex behavior in natural systems declined, leading to a deficit in published literature surrounding same‐sex behaviors for nearly a decade (Anderson et al. 2024; Ham et al. 2023).

One of the next instances where same‐sex behaviors appeared in the literature was Anne Innes Dagg's “Homosexual behavior and female‐male mounting in mammals—a first survey” published in 1984. Dagg's work was the first major survey and review of homosexual behaviors across taxa (Brooks 2021), laying the foundation for future synthesis by the likes of Roughgarden and Baghemil in the 1990s and early 2000s. Same‐sex behaviors are now known to occur in > 1500 species, but behaviors that do not fit neatly into a cis‐heteronormative framework still suffer from contemporary publication bias, with researchers citing the rareness and irrelevance of queerness as a reason against publication and funding (Anderson et al. 2024; Means and Morgenroth 2024).

By the 1980s, the AIDS epidemic unleashed unprecedented devastation on LGBTQ+ communities, marking a turning point in both social history and the trajectory of scientific inquiry into sexuality. The first case of AIDS, originally called GRID (Gay‐Related Immune Deficiency), in the US was reported in 1981. President Ronald Reagan did not publicly acknowledge the epidemic until 1985, indicative of the public marginalization and neglect of the gay community. Even while the gay community suffered from the AIDS epidemic, they remained the center of scientists' inquiries. During this period, psychologists such as Michael Ruse and J.D. Weinrich posited that in order to offset the genetic disadvantage to homosexuality, homosexual individuals must have increased abilities and intelligence (Ruse 1981; Weinrich 1978). When these searches failed to yield results, the brains of deceased men from the AIDS epidemic were examined to instead determine a biological basis for homosexuality (LeVay 1991; Swaab et al. 1995; Swaab and Hofman 1990).

As sequencing technology advanced in the 1990s, biologists doubled down on extensive searches for “gay genes” and “biological substrate” to rationalize queer behavior as heritable (Hamer et al. 1993; LeVay 1991; Levay and Hamer 1994; Swaab and Hofman 1990). Levay and Hamer (1994) used gay brothers to attempt to identify a correlation between homosexuality and the presence of a specific genetic marker (Levay and Hamer 1994), which was later scrutinized for subject selection bias (Marshall 1995). Although the 1980s and 1990s brought extreme loss of life within the gay and queer communities and heightened biological and genetic scrutiny from EEB and medical fields, LGBTQ+ activism through organizations like AIDS Coalition to Unleash Power (ACT UP) brought unity and a sense of belonging to the community through civil disobedience.

Scientific interest has since largely shifted to probing for explanations of queerness, mirroring the broader context of societal heteronormativity. Some researchers have proposed that same‐sex sexuality is a byproduct of selection for a separate adaptive trait, whereas other researchers have postulated that an individual engaging in same‐sex behavior is doing so because they mistakenly believe their partner is of the opposite sex (Bagemihl 1999; Poiani 2010; Sommer and Vasey 2006). Recent evolutionary literature demonstrates this pattern. Persistent debate has surrounded the phylogenetic origins of same‐sex behaviors (Clive et al. 2020; Kamath et al. 2020; Monk et al. 2019), with the argument that same‐sex behaviors are a phylogenetically ubiquitous ancestral condition stemming from non‐discriminate sexual mating (Monk et al. 2019). Challenges to this theory argue for further testing of both adaptive and non‐adaptive theories (Clive et al. 2020; Lerch and Servedio 2025). This more recent research brings us to a contemporary space, where we now must evaluate the contemporary relationship between science, society, and the LGBTQ+ community (see *Principle 5* and *6* for further discussion).

### 2.2 Contemporary Relationship Between Society, Science, and the LGBTQ+ Community

In the current moment, it is especially urgent to recognize that queer history does not represent a simple linear increase of tolerance and liberation, but it is a history where harsh setbacks to progress continually led to the loss of life, acceptance, and scientific knowledge. The contemporary relationship between scientific research and LGBTQ+ liberation remains as complex and multifaceted as it has been throughout history. Queer activists hold varied perspectives on genetic and biological research, with some viewing it as providing rhetorical support for liberation, and others as a potential basis for oppression (Wuest 2023). This complexity reflects the reciprocal and co‐constructive dynamic between science and society, where scientific frameworks both shape and are shaped by societal and political contexts.

This relationship between science and policy has become increasingly fraught, and misinformation about science is being leveraged to support political agendas (Miyagi et al. 2021; Rice et al. 2025; Sharpe et al. 2023; Sudai et al. 2022). Although this is not an exclusively contemporary phenomenon, it has a particularly acute impact in the current day. For instance, an NSF‐funded and disseminated human behavior educational project titled “Man: A Course of Study” reached national‐level controversy in 1975 for its “distortion of basic family values” as described by Rep. John Conlan (R ‐ AZ). This criticism resulted in a proposal in the House of Representatives that would require all NSF projects to gain final approval by Congress before being funded, which eventually failed (NSF 2025). Despite the historical precedent of such political reactions to scientific work, scientists still ignore, intentionally or unintentionally, the downstream implications their work can have when misused in politics (as in McNutt 2024). Ultimately, harm occurs when scientific research is misused to justify regressive and oppressive laws (Sudai et al. 2022), deny human rights and healthcare, and harm LGBTQ+ individuals.

In 2025, the Trump administration issued two executive orders with profound implications for both the scientific community and LGBTQ+ individuals. Executive Order 14168, “Defending Women from Gender Ideology Extremism and Restoring Biological Truth to the Federal Government” (The White House 2025a) leverages an oversimplified “biological” definition of sex to exclude TGNCI individuals. This executive order demonstrates how misinformation and misuse of science can have direct impacts on people and communities (Sudai et al. 2022). Executive Order 14173, “Ending Illegal Discrimination and Restoring Merit‐Based Opportunity” (The White House 2025b), is explicitly designed to dismantle resources for TGNCI individuals and diversity, equity, and inclusion efforts nationwide. These orders created a crisis affecting both scientific research and LGBTQ+ communities (and especially affected scientists who are TGNCI).

Implementation of these executive orders triggered immediate and sweeping changes across federal scientific agencies. The Centers for Disease Control and Prevention (CDC) instructed its scientists to pause and retract any publications containing terms like “LGBT,” “gender,” “transgender,” and “nonbinary,” while also removing mentions of HIV from its webpage (ACLU 2025; Mueller 2025). Similar erasure of LGBTQ+ focused research occurred at the National Institutes of Health (NIH), Department of Homeland Security (DHS), and Food and Drug Administration (FDA). Even the National Parks Service erased the words “transgender” and “queer” from the Stonewall National Monument (a monument dedicated to queer liberation) website (Moore 2025), stemming from the latest pushback by the federal government on trans rights (Kim 2025). The ripple effects also extended to the NSF and NIH, which paused all grant reviews in response to these orders, effectively halting the funding pipeline for countless research projects across the US and internationally. Professional organizations like the American Society for Microbiology, which has more than 37,000 members, have removed resources for LGBTQ+ people from their website, demonstrating how quickly scientific institutions responded to political pressure by erasing content supporting vulnerable communities. These actions have directly impacted scientists in countless ways (American Society of Naturalists 2025), including disrupting the dissemination of peer‐reviewed scientific research and marginalizing LGBTQ+ scientists who conduct research in these fields.

As public funding for science becomes increasingly politicized and restricted, private interests gain more influence in determining which scientific questions get answered and which research directions receive support (Crew 2025; Ro 2025). It is worth also noting that with the proposed scale of losses in federal funding, it cannot be expected that private funding will be able to close the gap (Diamond Jr. 1999; National Bureau of Economic Research 2021). This shift in science funding landscapes is further complicated by the explicit targeting of LGBTQ+ research and LGBTQ+ researchers with “banned words” and policies that prohibit terms related to gender and sexuality (among other minoritized identities) (J. Mervis 2025). These bans are not limited to research on the LGBTQ+ community, but affect grants across disciplines, including fields of science, health, technology, and more. These acts undermine the objectivity, independence, and diversity of scientific inquiry, limiting research that benefits both marginalized communities and all of society.

## 3 Where We Go From Here: Queering Ecology and Evolutionary Biology

Science, in all its senses, is a social process that both causes and is caused by social organization. To do science is to be a social actor engaged, whether one likes it or not, in political activity.—Richard Levins & Richard Lewontin, *The Dialectical Biologist* (1985)

Queering ecology and evolutionary biology is not simply about representation or inclusion; indeed, our aim is not to be included in a racist, cis‐hetero, and patriarchal structure, but rather to transform the very foundations of how knowledge is produced, taught, and allowed to reverberate through society. It is also a commitment to practices that recognize how the relationship between science and society, as well as systems of power, have impacted foundational knowledge in the field. However, understanding the reciprocal relationship between science and society, and between EEB and the LGBTQ+ community, is only the first step in transforming the field. To move forward, we must dismantle exclusionary logics embedded in scientific thought, confront biases, reclaim suppressed knowledge(s) of biological diversity, and commit to politically responsible science that resists being weaponized against vulnerable communities.

**FIGURE 1:**
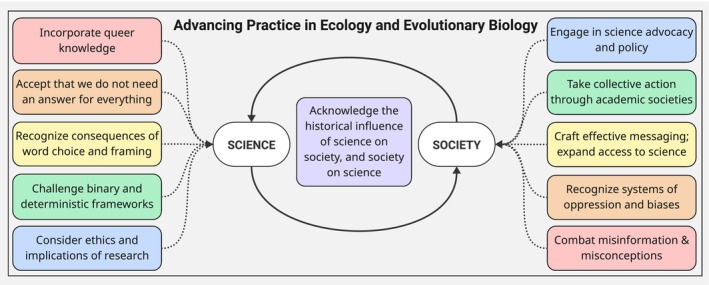
Proposed framework and action items for queering ecology and evolutionary biology and the impacts on the science and society feedback loop.

We envision that by recognizing the intersection between systems of oppression, incorporating queer knowledge into scientific thought, challenging biased (e.g., binary, deterministic) frameworks, choosing inclusive terminology, understanding that hypothesis‐framing matters, considering ethics and political implications when developing lines of research inquiry, and accepting that we do not need to have an answer for everything, we can actively queer EEB and advance better science (Figure 1). In this way, queering EEB is not just about inclusion; it is an epistemological intervention that expands the conceptual boundaries of what counts as scientific knowledge (Estien 2025; Mortimer‐Sandilands and Erickson 2010; Morton 2010; Vakoch 2020). Leveraging our framework for queering ecology and evolutionary biology (Figure 1), we present six principles and associated actionable items for scientists to move forward with ethical consideration of LGBTQ+ rights.

### 3.1 Principle 1. Recognize the Intersection Between Systems of Oppression

Systems of oppression do not operate in isolation, but intersect and reinforce one another in scientific discourse and practice. In fact, the history of scientific literature demonstrates how racial typology and “othering” have directly influenced anti‐queerness in science for centuries (Somerville 1994). This interconnected relationship necessitates an approach that recognizes how intersecting oppression shapes both knowledge production and scientific communities.

For example, Traditional Ecological Knowledge and Two‐Spirit ontologies—Indigenous queer ways of knowing—illuminate ecological dynamics (e.g., non‐monogamous mating systems, cooperative parenting, interspecies kinship) frequently dismissed in Western scientific paradigms, which are narrowly focused on reproductive fitness (TallBear and Willey 2019). Similarly, Black feminist theorists of marine biology have critically examined how even supposedly “neutral” language of field guides encodes hierarchies of race, gender, and sexuality, marking animals and behaviors outside the dominant frame as deviant, vagrant, or failed (Gumbs 2020).

These critiques reveal structural patterns in how systems of oppression influence the production and validation of scientific knowledge. When we recognize these patterns, we can transform practice to be more accurate and inclusive. By realizing systems of oppression are intersectional, we can then interrogate who has access to education, training, employment, outreach, and funding. Even if a person from a marginalized background ends up in science, they can still feel out of place because of biases and lack of access (e.g., Nguyen et al. 2022). The solution to resolving this bias lies in expanding access to science for all.

#### 3.1.1 Action: Consider Who Does, and Who Does Not, Have Access to Science

Although much of science outreach aims to reach underserved or marginalized communities, certain demographics (e.g., urban or rural communities) lack the same access to opportunity. To combat this, scientists should strive to actively, thoughtfully, and creatively reach audiences that are traditionally not reached by outreach efforts. When seeking to expand outreach, scientists can consider reaching these underserved communities, local businesses, and arts associations, or targeting community‐oriented professionals in industries like beauty, healthcare, and trade.

Innovative and conversational ideas for expanding outreach can look like: public facing galleries, a social hour connected to academic presentations such as lectures on tap (https://lecturesontap.com/), ecological or environmental experiences (e.g., guided hikes, tidepooling, or snorkeling), and community science (e.g., Bioblitz on iNatualist in local parks and trails). These events can and should be done in multiple languages. They can be transdisciplinary and interdisciplinary (e.g., incorporating art, music, or literature) to move away from a unidirectional transmission of information to a more collaborative and interactive conversation. Examples like “Climate Chisme” (climate gossip) by Queer Brown Vegans demonstrate how culturally specific approaches can reach new audiences through familiar formats like podcasts. A key to expanding access is to engage deeper in 1‐on‐1 interactions that start by sharing common ground using this as a starting point for meaningful scientific dialogue.

### 3.2 Principle 2. Incorporate Queer Knowledge Into Scientific Thought

Incorporating queer lived experiences into scientific thought is the foundation of an epistemological intervention. Although personal thoughts, feelings, and perspectives are not scientific observations themselves, the unique lens through which LGBTQ+ scientists view the natural world (e.g., biological variation and behavior; Adsit‐Morris and Gough 2021; D. J. Haraway 2016; Hazard 2024; McLaughlin et al. 2023; Seymour 2020) can enhance scientific practice by illuminating cis‐heteronormative assumptions that have historically shaped scientific narratives (e.g., Box 1). Additionally, queer ecology can challenge cis‐heteronormative assumptions embedded in ecological and evolutionary narratives by taking a diversity‐affirming approach (see Roughgarden 2004). By incorporating queer lived experiences, EEB scientists are invited into the LGBTQ+ community's way of thinking and knowing, enhancing scientific rigor through understanding meaningful biological variation.

#### 3.2.1 Action: Building Support Within the Scientific Community

To incorporate queer perspectives into scientific thought, we must create safe spaces for LGBTQ+ scientists in EEB. Even when faced with limited funding due to societal and political impacts, inclusion of varied perspectives, including LGBTQ+ identities, should be maintained as a priority for EEB. Queering ecology and evolution requires the participation and attention of all EEB researchers. Here, we highlight some successful initiatives that have helped to create an equitable and inclusive environment for LGBTQ+ researchers and their colleagues.

At the academic society level, attention should be dedicated to conferring upon and disseminating changes to standards, particularly around word choice and framing (e.g., species nomenclature, as done by the American Ornithological Society Leadership 2023). For example, The Society for Integrative and Comparative Biology organized a symposium “Sexual Diversity and Variation” at their 2023 annual meeting to address the question “Can sexual diversity be accurately captured by a male‐female binary?” (Lewis and Sharpe 2023). A roundtable talk featured intersex activists and biologists who shared their work on sex and gender concepts, resulting in a compiled list of recommended resources for inclusive biology education (Sharpe et al. 2023). A key thematic takeaway from the symposium was to encourage specificity when discussing biological sex‐related terms in research, a principle also highlighted in this manuscript.

Societies can also prioritize group‐oriented affinity events at their conferences to provide important spaces for community. For example, the Tri‐Societies (Society for the Study of Evolution, American Society of Naturalists, and Society of Systematic Biologists) Evolution Conference hosted Evolution's Rainbow in 2022, an event which combined talks and discussions on sex, gender, and sexuality in nature, hosted by drag queen Nina West. The event culminated with a panel discussion on how the EEB community can better support and retain LGBTQ+ students and colleagues. The conference also coordinated a LGBTQ+ networking lunch, which paired self‐identified LGBTQ+ conference attendees in small groups of 4–6 for a 2‐h lunch break to network and discuss aspects of the queer experience in EEB. Although running annual events such as Evolution's Rainbow can be time‐consuming and cost‐prohibitive, providing a platform for conference attendees to arrange networking events with scientists with similar identities is free to societies and should be considered for regular inclusion in conference programming.

### 3.3 Principle 3. Challenge Binary and Essentialist Frameworks

Evolutionary theory has historically been shaped by binary and deterministic frameworks that fail to capture the multivariate and non‐linear nature of some biological processes (Box 1). Queer theory invites us to teach and investigate biology in ways that acknowledge the multiplicity of species behaviors—behaviors that routinely exceed, contradict, or exist entirely outside the normative boundaries of compulsory cis‐hetero‐monogamy (e.g., self‐fertilization, multiple partners, sex changes, and more).

Cooperation, in particular, is a central evolutionary force that offers an alternative to competition‐dominated models. Roughgarden and colleagues have argued that classic sexual selection is incomplete and that social selection helps explain cooperation, coalition building, and mating systems beyond competition alone (Roughgarden 2004; West‐Eberhard 1983); cooperation can increase fitness through kin selection, mutualism, reciprocity, and, in some cases, multi‐level selection (Roughgarden 2004; Trivers 1971; Wilson and Wilson 2007). Other behaviors that exist outside of normative boundaries include same‐sex behavior, pair bonding, alloparenting, and social role flexibility, which can stabilize group dynamics, improve access to resources, and ultimately affect reproductive success. Bonobos, among our closest living relatives, show female‐centered hierarchies and frequent same‐sex sociosexual behavior that maintains alliances, reduces conflict, and supports infant care (Sommer and Vasey 2006; Surbeck et al. 2025). Many reef fishes, such as wrasses and clownfish, exhibit sequential hermaphroditism, altering sex in response to environmental and social cues (Bagemihl 1999). Birds such as albatrosses, gulls, and swans have documented same‐sex pairings that defend territories and, at times, raise offspring through cooperative arrangements (Bagemihl 1999; Hunt and Hunt 1977; Young and VanderWerf 2014). Numerous plants exhibit self‐fertilization, parthenogenesis, and mixed mating systems (Subramaniam and Bartlett 2023), whereas many vertebrates engage in multiple mating and cooperative breeding systems (Cockburn 1998; Hatchwell 2009; Wanders et al. 2023). Inclusion of behaviors, reproductive roles, and social hierarchies which fall outside of a gendered and binary “norm” enhances our understanding of the diversity of roles in natural systems.

#### 3.3.1 Action: Combating Misinformation and Misconceptions Through Education

In EEB classrooms, instructor decisions about how topics will be taught are not neutral. Racism, homophobia, and transphobia flourish when unchallenged in classrooms (Bickford 2022). A comprehensive review of 153 classroom biology found that textbooks routinely omit or misrepresent evidence on race, sexuality, and intersex variation under political and economic pressures, and virtually none discuss non‐procreative sex or nonbinary biological sex realities (Bickford 2022). Further, it is known that students, especially those who are LGBTQ+, are negatively impacted when instructors inadequately teach or avoid teaching these topics. When instructors fail to explain the diversity of biological sex variation in classrooms, or remain silent about it, queer and trans students experience reduced connection to the course content and emotionally distance themselves from instructors (Casper et al. 2022). To address this, inclusion of small disclaimers, such as a statement like “sex and gender are more expansive topics than what we are able to cover today”, can improve queer and trans student experiences (Casper et al. 2022). Further, including succinct natural examples of the biological diversity of sex variation is a scientifically accurate and non‐disputable way to address this topic.

Other biases that are rooted in deterministic or essentialist frameworks still persist in the classroom. For instance, one study found that after an introductory‐level biology course, the majority of students conflate genetic drift, a random process, with selection, a non‐random process (Andrews et al. 2012; Carlen, Chacko, et al. 2025). In this case, students leave the classroom with the expectation that evolution is solely deterministic, a harmful way of thinking that can be related to the idea that queerness has a genetic underpinning that is hardwired and thus can be “fixed” (Carlen, Chacko, et al. 2025).

A plethora of new resources for instructors are increasingly being developed to support a more inclusive and accurate teaching effort, such as lesson plans, syllabi, and terminology guides. Resources include *Gender Inclusive Biology* (genderinclusivebiology.com), a repository of science curriculum with ready‐to‐use lesson plans, educator toolkits, and professional development materials that inclusively address gender and sexuality in the classroom, and *Project Biodiversify* (projectbiodiversify.org), which aims to expose biology students to a diversity of role models in the field of biology through teaching materials and workshops, and more (see Carlen, Chacko, et al. 2025; Costello et al. 2024; Moreau et al. 2022; Zemenick et al. 2022). Beyond improving teaching methodologies, misconceptions can be retroactively addressed with coursework that directly evaluates the historic precedent of bias in EEB research (e.g., Science Behind Bias Seminar Course; Moreau et al. 2022; Renn 2010).

### 3.4 Principle 4. Understand That Language and Hypothesis‐Framing Matter

Scientific terminology and hypotheses are not value‐neutral when read beyond scientific audiences. Language carries significant social and political weight, shaping not only how findings are communicated but also how biological and social realities are understood (Rice et al. 2025; Trisos et al. 2021). Hypotheses in EEB are often framed in “paradoxical” ways that make sense within scientific discourse, but can be misinterpreted by non‐scientific audiences. For instance, questions about the “evolutionary paradox” of same‐sex mating may seem like neutral inquiries to scientists, but implicitly frame non‐reproductive sexual behaviors as abnormal and require investigation. This, in turn, produces the very value judgments that scientists may actually be aiming to deconstruct. Scientists must therefore critically examine how we frame hypotheses and choose terminology; doing so not only benefits our scientific audience, but also helps us understand how these choices may be interpreted in other contexts (e.g., socially or politically). The solution lies in scientists clearly communicating the real‐world implications of research and writing with awareness of how language might be received by broad audiences beyond academia.

#### 3.4.1 Action: Craft Effective Messaging

Although 89% of US adults consider scientists to be intelligent, just 45% of them think that scientists are effective communicators (Tyson 2024), a decrease of 9% since 2019 (Funk and Hefferon 2019). The solution to this is activism and clear communication, which can improve the credibility of scientists and their research (van Eck et al. 2024). To better craft effective and engaging public‐facing messages, scientists can leverage established communication practices. For example, scientists can learn from storytelling practices that center on resonance, memory, use descriptive imagery, or even humor to better engage audiences (Fernández‐Llamazares and Cabeza 2018; Green et al. 2018). Several professional development organizations teach these types of skills in workshops, such as the American Institute of Biological Sciences Communication Bootcamp (aibs.org), American Association for the Advancement of Science (AAAS) Communicating Science Seminars (aaas.org), and ComSciCon workshops (comscicon.org) for graduate students. Ecology and evolutionary biologists who are well‐trained in science communication and media can register with AAAS SciLine (sciline.org), a service that connects journalists with scientific experts to ensure that accurate information about biological research reaches the public through media channels.

### 3.5 Principle 5: Engage in Policy, Advocacy, and Collective Action

The refusal to engage with the societal and political implications of science is itself a political stance. A pillar of our framework is recognizing that scientific inquiry is inseparable from the societal and political conditions that enable and constrain it. Engagement can take multiple forms, from individual advocacy to collective action through professional societies. When necessary, scientists should consider how reframing messaging can keep critical science moving and accomplish pre‐existing goals before additional barriers were introduced (Welch 2024).

#### 3.5.1 Action: Accurately and Responsibly Inform Policy

Scientists have a unique responsibility to inform policy decisions with evidence‐based insights. When research findings have implications for society, scientists should engage with policymakers to ensure that the generated knowledge informs legislation and policy. By serving as experts and testifying or participating in advisory meetings, scientists bring necessary perspectives to these discussions. In addition to providing information, accurate communication with policymakers helps to cut through the noise of misinformation about science in society.

#### 3.5.2 Action: Take Collective Action Through Academic Societies

Academic societies can amplify individual voices through coordinated advocacy campaigns (Ryan et al. 2025). For example, societies can leverage their credibility to clarify the field's stance on a topic (e.g., the definition of biological sex; Eppley et al. 2026), improve the perception of trustworthiness of scientists, and advocate for scientific funding (Cheng et al. 2023; Ryan et al. 2025). Societies can establish public policy committees to handle oversight of these topics and organize initiatives like writing letters to Congress, developing policy position statements and education materials, and connecting society members with resources to do individual‐level advocacy. Several societies, such as the American Institute of Biological Sciences, the Ecological Society of America, and the Society for the Study of Evolution, already coordinate efforts like organizing Capitol Hill days and facilitate local district meetings between scientists and House of Representative members. These societies and others also offer public policy fellowships and training programs that provide members the opportunity to develop advocacy skills.

### 3.6 Principle 6. Accept That We Do Not Need to Have to an Answer for Everything

We emphasize that science does not need to have an answer for everything. Some questions resist black‐and‐white solutions, and in many cases ethical considerations require scientists to stop pursuing certain lines of inquiry altogether. No question is inherently bad (scientific questions in themselves can be value‐neutral), but it is the framing of various questions and the implications of potential answers that garner concern. Queering EEB means recognizing and accepting that sometimes answers fall outside traditional boundaries, and that ambiguity and refusal to neatly resolve tensions are themselves valuable. Many topics in science are hotly debated and we contend that it is not necessary to have a consensus (Eppley et al. 2026). For questions with particularly harmful implications, it is both acceptable and necessary to resist the desire to have an answer or land on a single definitive one.

#### 3.6.1 Action: Consider the Ethical Implications of Research

One recent example of potentially harmful inquiry is research on the phylogenetic and ancestral origins of same‐sex behavior, which have involved testing of theory, models, genetics, and behavior (e.g., Lerch and Servedio 2025; Monk et al. 2019, among others). Although these questions may be scientifically value‐neutral and the results of interest to scientific and LGBTQ+ communities, the potential implications of these findings still must be considered—what if a proximate heritable cause of same‐sex behavior is uncovered? Considering the historic context of how society has used scientific findings to carry out eugenic practices (e.g., sterilization, euthanisia, genocide), is continuing this line of inquiry ethical, especially in the current societal and political landscape? Is there potential for misuse?

## 4 Conclusions

Nature has never fit neatly into our boxes, yet science has long tried to contain it within frameworks that exclude diverse ways of knowing. As EEB scientists working in an era of rapid societal, scientific, and global change (including increasing misinformation, systematic dismantling of science, and threats to LGBTQ+ safety), we must consider how interactions between science and society have historically influenced each other, and how our individual actions and roles continue to contribute to this cycle. Diverse ways of knowing can help us navigate this era of rapid change.

In this paper, we have drawn on our queer lived experiences to outline six principles to confront existing biases and produce better science: (1) recognize intersections between systems of oppression; (2) incorporate queer lived experiences into scientific thought; (3) challenge biased (e.g., binary, deterministic) frameworks; (4) understand that language and hypothesis framing shape both science and its societal interpretation; (5) embed ethics and political responsibility in the development of research inquiries; (6) accept that we do not need answers for everything. By integrating these principles within mainstream scientific practice, we can strengthen the reciprocal relationship between science and society, where advances in one transform the other, collectively moving towards knowledge that is more accurate, reproducible, and inclusive.

## Author Contributions

**Madeline G. Eppley:** conceptualization (lead), investigation (lead), project administration (lead), visualization (equal), writing – original draft (lead), writing – review and editing (lead). **Robert J. Dellinger:** investigation (equal), visualization (equal), writing – original draft (lead), writing – review and editing (lead). **Leila Curtis:** investigation (equal), writing – original draft (supporting), writing – review and editing (supporting). **Cesar O. Estien:** investigation (equal), writing – original draft (supporting), writing – review and editing (supporting). **Lindsey Forg:** investigation (equal), visualization (equal), writing – original draft (supporting), writing – review and editing (supporting). **Angela J. Jones:** investigation (equal), writing – original draft (supporting), writing – review and editing (supporting). **Ally Swank:** conceptualization (lead), investigation (lead), visualization (equal), writing – original draft (lead), writing – review and editing (lead). **Andy Lee:** conceptualization (lead), investigation (lead), visualization (equal), writing – original draft (lead), writing – review and editing (lead).

## Disclosure

Positionality statement of the authors: The authors of this paper are a group of early‐career and openly queer (LGBTQ+) authors, including queer, transgender, agender, nonbinary, genderqueer, gay, asexual, polyamorous, and other identities. We are an intersectional group of undergraduate and graduate students across predominantly white institutions in the United States of America (US). Some of the authors also hold identities (individually and in combination) as disabled, immigrant, neurodivergent, low‐income, people of color from many ethnic backgrounds, and first‐generation academics. We recognize that our perspectives in this paper are informed by our personal experiences, backgrounds, and identities. We felt that it was timely and important to share our experiences to help facilitate a safer environment for future scientists. We also recognize the ongoing evolution of the field and emphasize that our perspectives are reflective of the current climate.

## Conflicts of Interest

The authors declare no conflicts of interest.

## Data Availability

No data were collected as part of this research.
